# Multi-membership gene regulation in pathway based microarray analysis

**DOI:** 10.1186/1748-7188-6-22

**Published:** 2011-09-22

**Authors:** Stelios P Pavlidis, Annette M Payne, Stephen M Swift

**Affiliations:** 1 School of Information Systems, Computing and Maths, Brunel University, Uxbridge, UB8 3PH, UK

## Abstract

**Background:**

Gene expression analysis has been intensively researched for more than a decade. Recently, there has been elevated interest in the integration of microarray data analysis with other types of biological knowledge in a holistic analytical approach. We propose a methodology that can be facilitated for pathway based microarray data analysis, based on the observation that a substantial proportion of genes present in biochemical pathway databases are members of a number of distinct pathways. Our methodology aims towards establishing the state of individual pathways, by identifying those truly affected by the experimental conditions based on the behaviour of such genes. For that purpose it considers all the pathways in which a gene participates and the general census of gene expression per pathway.

**Results:**

We utilise hill climbing, simulated annealing and a genetic algorithm to analyse the consistency of the produced results, through the application of fuzzy adjusted rand indexes and hamming distance. All algorithms produce highly consistent genes to pathways allocations, revealing the contribution of genes to pathway functionality, in agreement with current pathway state visualisation techniques, with the simulated annealing search proving slightly superior in terms of efficiency.

**Conclusions:**

We show that the expression values of genes, which are members of a number of biochemical pathways or modules, are the net effect of the contribution of each gene to these biochemical processes. We show that by manipulating the pathway and module contribution of such genes to follow underlying trends we can interpret microarray results centred on the behaviour of these genes.

## Background

Pathway based microarray data analysis is an attempt to integrate microarray data analysis with biochemical pathway knowledge [1]. Rather than concentrating on the often subtle change occurring in the expression of individual genes, gene expression analysis is facilitated to identify coordinated changes occurring in the expression of sets of genes, forming biochemical pathways [2]. The ultimate goal of this approach is to decipher the functional state of a cell at the level of the underlying biochemistry.

Biochemical pathway data is readily accessible in various public databases, such as KEGG [3], Reactome [4], SABIO-RK [5], EcoCyc [6] and others, while tools developed for visualisation of genes' behaviour, based on microarray data, include Eu.Gene [2], GenMapp [7], Cytoscape [8], Pathfinder [9], GeneNet [10] and GScope [11]. These software tools are based on superimposing a single microarray dataset on a biochemical pathway database, in order to visualise the expression of each individual gene per pathway and thus establish the state of individual pathways.

However, genes in a biochemical pathway often show quite variable behaviour in terms of RNA production and previous work in the field has already suggested that not all such genes are representative of the pathway's behaviour [12]. To an extent this is a consequence of the fact that genes forming a pathway may encode proteins of very different functionality with some being transcription factors acting in the cell nucleus while others proteins residing on the cell membrane [13]. Additionally the existence of different levels of regulation, including translation, protein maturation and degradation rate, may confer gene expression insufficient evidence of gene functionality [14,15]. Notably, microarray analysis itself is accompanied by limitations, as it involves numerous error-prone experimental steps and requires the physical disruption of cells to gain access to their gene expression patterns [16].

**Figure 1:**
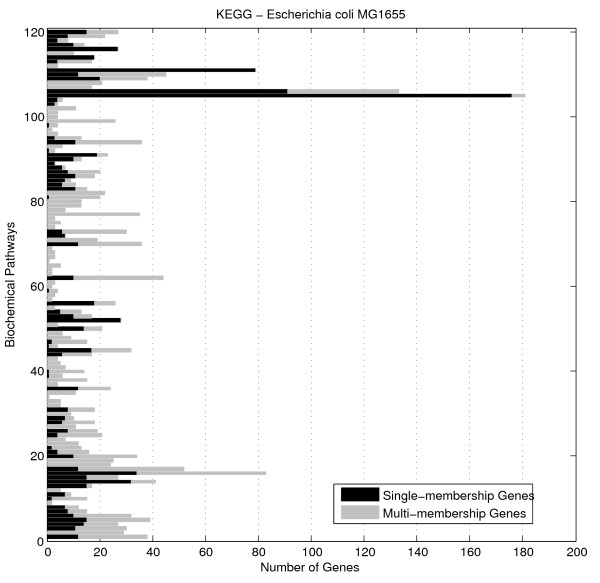
**Number of single and multi-membership genes constituting each MG1655 *Escherichia coli *KEGG pathway**. Single-membership genes are represented in black, while multi-membership genes in lighter grey colour. Evidently, genes of multi-membership nature are present in significant proportion in most biochemical pathways.

We however, have suggested an additional cause for observing variation in the expression of genes, forming a biochemical pathway. According to the Kyoto encyclopaedia of genes and genomes database (KEGG), it is quite common for a gene to be a member of two or more biochemical pathways. We refer to such genes as multi-membership genes to distinct them from single-membership genes that are members of one and only pathway. Figure 1 reveals the number of single and multi-membership genes forming each of the *Escherichia coli *K12 pathways, currently stored in KEGG database.

According to our hypothesis, differential expression of a multi-membership gene may be the effect of different regulation of that gene, by the biological system, at the level of transcription due to its involvement in the activity of more than one biochemical pathways. Consequentially, the observed expression of any such gene corresponds to the net effect of its regulation in order to contribute adequately to the activity of the biochemical pathways it is a member of. To our knowledge the multi-membership nature of genes and its impact on pathway based microarray analysis has not been extensively explored. This is an omission that can potentially lead to misleading conclusions, given that multi-membership genes may mask the true behaviour of individual pathways.

We assume that an increase or a decrease in the activity of a biochemical pathway is accompanied by a respective trend for increase or decrease in the functionality of most of the genes forming the pathway, whose fluctuations in expression affect the given pathways' state. Thus, to the extent that gene functionality is adjusted at the level of transcription, we expect genes to be regulated by the living system in a way that follows the trend of expression in a particular pathway and to generally show consistent behaviour, with only few exceptions, such as repressor genes and alternative isoenzymes.

We have proposed a methodology that takes into account the expression of all genes in a given organism, that are members of biochemical pathways, and the consensus of gene expression per pathway in order to identify the underlying pathway expression changes caused by the biological system through regulation of the expression of their constituent genes [17]. Unlike other approaches where genes are treated as stable or differentially expressed, our methodology considers the up- or down-regulated state of each individual gene. We attempt to ascribe any observed inconsistencies in gene expression in a pathway, to the involvement of some of its genes in the activity of other pathways of which they are also members. We implemented a hill climbing search approach [18], which was able to produce consistent results, in agreement with the publications accompanying the data in question.

Given the tendency of the hill climbing search to get trapped into local maxima, we proceeded further, applying a simulated annealing [19] and a genetic algorithm [20] search technique in order to explore the performance of each one on a set of microarray experiments. Since differences in the final fitness reached by each of these methods do not have a straightforward biological meaning we shifted our efforts towards exploring the similarity of the produced results, in conjunction with their corresponding fitness, by using two complimentary approaches. In particular, we developed a methodology for estimating the similarity of two gene allocations in terms of the probability of obtaining them purely by chance. Additionally, we adopted the fuzzy adjusted rand index metric, widely used measure of agreement for categorical data [21]. Interestingly, according to both measures the results produced by all methods are highly consistent, while the simulated annealing search appears to be only slightly more efficient than the hill climbing and genetic algorithm techniques.

In addition, we further developed the simulated annealing approach to work with modules, that is, shorter chains of biochemical events, which form part of KEGG pathways, applying the methodology to a number of microarray experiments.

## Methods

Microarray datasets are trimmed to only include genes present in KEGG pathways. For example, KEGG contains 1384 *Escherichia coli *genes out of a total of 4288 protein-coding genes, for the harmless laboratory strain K12 [22]. We apply discretisation of genes into three categories, namely up-, down-regulated and stable, based on an adequately chosen threshold and apply a hill climbing, a simulated annealing and a genetic algorithm, to alter the possible allocation of multi-membership genes to their constituent pathways.

We assume that a differentially expressed gene is regulated by the biological system to contribute to the activity of at least one of the pathways it is a member of. Thus, any configuration that satisfies this criterion is considered valid, while an allocation where a multi-membership gene has not been assigned to any of its pathways is rejected. We attempt to identify for each multi-membership gene the pathways whose activity requires the contribution of that gene and the direction of regulation required by each one those pathways. Allocation of a gene to one of its constituent pathways suggests that the biological system has adjusted the expression of that gene in the given manner to satisfy the activity of that pathway. Naturally, not allocating a gene to a pathway suggests its expression is not related to its involvement in the activity of this particular pathway.

Given that KEGG pathways contain collections of genes involved in extensive biochemical processes, we further proceed to work with KEGG modules, following a similar rationale to the one applied in the case of pathways.

### Notation

We use the following mathematical notation in our methods and results. Let *P *be an *N *row by *M *column binary matrix, *P∈ *{0,1}*^N×M^*, where *p_ij _*(the element in the *i*th row and *j*th column of matrix *P*) is equal to 1 if gene *i *is a member of pathway *j*, and *p_ij _*= 0 if gene *i *is not a member of pathway *j*. Therefore *P *represents a snapshot of the KEGG membership of genes to pathways for a given species and does not change.

Let *A *∈ {0,1}*^N×M ^*be a binary matrix such that *P-A *∈ {0,1}*^N×M^*, where *A *represents a potential allocation of genes to pathways that we use in our methods. Here *a^ij ^= *1 if gene *i *is allocated to pathway *j *and a*_ij _*= 0 if gene *i *is not allocated to pathway *j*. The restriction *P-A *∈ {0,1}*^N×M ^*means that *A *can define pathways to have less genes than originally in *P*, but can never have genes that contradict *P*, i.e. we do not allow allocations that would be contrary to the allocations in KEGG.

### Fitness Function

Hill climbing, simulated annealing and genetic algorithms are heuristic search methods and as such require a fitness function to be defined, which the algorithm attempts to maximise or minimise, depending on the type of the problem in hand [23]. A fitness function evaluates the worth of the current solution being considered by a method. The fitness function we implement evaluates the worth of an allocation of multi-membership genes to the pathways they are members of, and is used by all three heuristic search methods.

Let us assume that we have a single set of gene expression data (one experiment) for the *N *genes called *G*. We score an allocation on how much each pathway is down- or up-regulated according to equations (1) to (3). Note that the constant *c *is a threshold parameter defining the state of a gene in terms of up- and down-regulation. In particular, *X *(*i*) has a value of +1, -1 or 0 if gene *i *is up-, down-regulated or stable respectively.

(1) $$X\left(i\right)=\left\{\begin{array}{c} +1\\ -1\\ 0 \end{array}\right)\begin{array}{c} \text{,if }G\text{(}i\text{) }>\text{ c}\\ ,\text{if }G\text{(}i\text{) }<\text{ - c}\\ \text{,otherwise} \end{array}$$

(2) $$F\left(A\right)=\overset{M}{\underset{j=1}{\sum }}\left|\overset{N}{\underset{i=1}{\sum }}H\left(a_{ij}\right)\right|$$

(3) $$H\left(a_{ij}\right)=\left\{\begin{array}{c} X\left(i\right)\\ 0 \end{array}\right)\begin{array}{c} \text{,if }a_{\text{ij}}\;=\text{ 1}\\ ,\text{otherwise} \end{array}$$

*F*(*A*) is the fitness function, which we aim to maximise by changing the allocation of multi-membership genes to their corresponding pathways. We use equation (3) to define if gene *i *is a member of pathway *j*, which is true if *a_ij _*= 1, and if that is the case to define if the gene is up-, down-regulated or stable. Function $\overset{N}{\underset{i=1}{\sum }}H\left(a_{ij}\right)$reveals the difference between the numbers of up- and down-regulated genes in pathway *j*. Thus, the more genes of similar expression are allocated to pathway *j *the greater $\left|\overset{N}{\underset{i=1}{\sum }}H\left(a_{ij}\right)\right|$ becomes for that pathway, leading to an increase of the value of *F*(*A*).

### Hill Climbing

**Algorithm 1**, Hill Climbing Search Algorithm

1. INPUT: *a *= list of gene IDs coupled with their pathway IDs, *b *= expression vector of log2 ratios, *c *= threshold for up-/down-regulated genes

2. Remove all genes between +*c *and -*c*

3. Randomly allocate each expressed gene to its member pathways (create *A*)

4. Get fitness *F*(*A*), set *F_old *= *F*(*A*)

5. For *j *= 1: number of iterations

6. Save gene configuration

7. Use *P *to randomly choose a gene (*i*) with multiple membership and randomly

     choose one of the pathways(*j*) it belongs to

8. If according to *A *gene (*i*) is already present in the pathway (*j*) Then

9. Remove the gene, i.e. set *a_ij _*= 0

10. Else

11. If not present then place it in the pathway, i.e. set *a_ij _*= 1

12. End if

13. If the gene is not assigned to at least one pathway then randomly choose a pathway and assign it to it

14. Estimate fitness *F*(*A*)

15. If *F*(*A*) >*F_old *then

16. Set *F_old *= *F*(*A*)

17. Else

18. if *F*(*A*) <*F_old *then Restore gene configuration (step (6))

19. End if

20. End for

21. OUTPUT: *A*

### Simulated Annealing

In contrast to hill climbing, simulated annealing may accept a solution of lower fitness, depending on a probability which is defined by gradually decreasing parameter *T*, termed temperature. We have chosen a starting temperature *T *= 1 and a final temperature *T *= 0.01 as appropriate for 10000 iterations which have proven sufficient for the algorithm to converge. At step 15) of the hill climbing algorithm above, the simulated annealing approach accepts an allocation of lower fitness with a probability which can be estimated based on equations (4) to (6):

(4) $$P_{t}=e^{\frac{-\Delta F}{T_{t}}}$$

(5) $$T_{t}=T_{t-1}\lambda$$

(6) $$\lambda =e^{\frac{\log\left(T_{FINAL}\right)-\log\left(T_{0}\right)}{I}}$$

Where *P_t _*is the probability of accepting an allocation of lower fitness at the current iteration *t, -ΔF *is the difference between the current fitness and the one of the allocation at the previous iteration, *T_t _*is the current temperature and *T_FINAL_*the temperature at the last iteration, *λ *is a constant and *I *the number of iterations for the search to complete.

### Genetic Algorithm

The genetic algorithm simulates evolution, where the fittest individuals are more likely to survive. At each generation we apply crossovers and mutations, changing the allocation of multi-membership genes to their member pathways. Algorithm 2 represents the main body of the genetic algorithm.

**Algorithm 2**, Genetic Algorithm

1. INPUT: *a *= list of gene IDs coupled with their pathway IDs, *b *= expression vector of log2 ratios, *c *= threshold for up-/down-regulated genes

2. Remove all genes between +*c *and -*c*

3. Create 100 random Parent chromosomes

4. Get fitness *F *of each Parent chromosome

5. For *i *= 1: number of generations

6. For *j *= 1:number of individuals in Parent

7. Call mutation Algorithm with input Parent*_j_*

8. End for

9. Create a random list List of (number of Mutated)/2

10. For *j *= 1:(number of Mutated)/2

11. Call crossover Algorithm with input Mutated(List(j)), Mutated(List(j+1))

12. End for

13. Get fitness of each Mutated and Crossover chromosome

14. Concatenate Parent, Mutated and Crossover chromosomes and their corresponding fitness

15. Sort the resulting chromosomes and fitness according to the later.

16. Set Parent = first 100 chromosomes and Fitness = first 100 fitness values

17. End for

18. OUTPUT: Best Individual and Fitness

Algorithm 3 describes the crossover process, while algorithm 4 the mutation process, called at steps 7 and 11 of the main genetic algorithm script, respectively. Within Algorithm 3 the operator C = [A_1_,A_2_,...,A_x_,B_x+1_,B_x+2_,...B_N_] concatenates the lists A and B preserving order and sets C to be the result.

**Algorithm 3**, Crossover Algorithm

1. INPUT: Parent A and Parent B

2. Choose a random number *x *between 1 and length of Parent A

3. Set Crossover A = [A_1_,A_2_,...,A_x_,B_x+1_,B_x+2_,...B_N_]

4. Set Crossover B = [B_1_,B_2_,...,B_x_,A_x+1_,A_x+2_,...A_N_]

5. OUTPUT: Crossover A, Crossover B

**Algorithm 4**, Mutation Algorithm

1. INPUT: Parent*_i_*

2. Set Mutated = Parent*_i_*

3. For *k *= 1:length of Mutated*_i_*

4. Produce a random number *a *between 0 and 1

5. If *a *< 1/length(Mutated*_i_*) randomly choose a position *x *in Mutated*_i _*{k}

6. If Mutated*_i _*{k}(x) = = 1, Then Set Mutated{k}(x) = 0

7. Else

8. If Mutated*_i _*{k}(x) = = 0, Then Set Mutated {k}(x) = 1

9. While sum of Mutated{k} = = 0, go to 4

10. End if

11. End for

12. OUTPUT: Mutated

A generation consisting of a hundred individuals proved sufficient to reach the expected fitness over about four hundred generations.

### Hamming Distance

The Hamming Distance (*Hamm *below) measure reveals the similarity between two binary strings of the same length [24]. Thus, it was a natural choice of method to evaluate the consistency of genes to pathways allocations.

Let *D,E *∈ *B^N×M ^*be gene to pathway allocations, as described in the Notation section, such that *P-D *∈ *B^N×M ^*and *P-E *∈ *B^N×M^*, i.e. *D *and *E *are allocations of genes. Let the similarity between *D *and *E *be:

(7) $$S\left(D,E\right)=\frac{1}{NM}\overset{N}{\underset{i=1}{\sum }}\left(M-Hamm\left(D_{i},E_{i}\right)\right)$$

(8) $$Hamm\left(D_{i},E_{i}\right)=\left\{\begin{array}{c} 1\\ 0 \end{array}\right)\begin{array}{c} \text{,}D_{i}=E_{i}\\ ,\text{otherwise} \end{array}$$

where *D_i _*is the *i*th row of *D*.

To obtain a meaningful interpretation of the observed hamming distances, we developed a methodology to estimate the probability of obtaining any hamming distance between two allocations produced by our methods, purely by chance. Firstly, for any given multi-membership gene, we estimate the probability of observing each possible hamming distance between pairs of allocations. The method is based on estimating the number of possible valid binary strings representing the allocation of a multi-membership gene to the pathways it is a member of, according to KEGG database.

In the simplest case of a gene that is a member of two pathways, its allocation is represented by a string of two binary digits. As already mentioned, only solutions where the gene has been allocated to at least one of the pathways it belongs to are considered valid. Therefore, a string consisting solely of zeros is not accepted as a valid allocation. Consequentially, the square matrix on Table 1 represents all valid combinations of allocations, for a gene that is a member of two distinct biochemical pathways, giving rise to all possible hamming distances.

**Table 1:** Hamming Distances between two allocations of a gene member of two biochemical pathways

	Allocation 2		
**Allocation 1**	**01**	**10**	**11**

**01**	0	2	1

**10**	2	0	1

**11**	1	1	0

The table reveals all possible combinations of two allocations for a multi-membership gene, participating in two distinct biochemical pathways, with the corresponding hamming distance between the binary strings representing these allocations. A string of zeros is considered invalid allocation, as we assume that a differentially expressed gene is contributing to the activity of at least one of its member pathways.

The probability of observing any of the hamming distances on Table 1 is equal to the number of combinations producing each of the possible hamming distances, namely 0, 1 and 2, divided by the overall number of possible combinations.

Again in the simplest case of a gene member of two pathways we obtain the probabilities shown on Table 2. For a gene that is a member of any number of pathways, the number of such combinations for any given hamming distance between 0 and *r *can be estimated according Table 3.

**Table 2:** Probability of obtaining any hamming distance between two allocations of a gene member of two pathways

**Hamming Dist**.	0	1	2
**Probability **	0.333 (3/9)	0.444 (4/9)	0.222 (2/9)

Given the number of possible combinations (Table 1) of allocations for a gene member of two pathways, and the hamming distance between them, Table 2 shows the probability of obtaining each possible hamming distance, purely by chance.

**Table 3:** Number of combinations of pairs allocations of hamming distance from 0 to *r*

**Hamming Dist**.	Number of possible occurrences
0:	2*^n ^*- 1

1:	(2*^n^*- 1 - *n*)*n *+ *n*(*n *-1) = (2*^n ^*-2)*n*

2:	$\begin{array}{c} \left(2^{n}-1-\left(\begin{array}{c} n\\ 2 \end{array}\right)\right)\left(\begin{array}{c} n\\ 2 \end{array}\right)+\left(\begin{array}{c} n\\ 2 \end{array}\right)\left(\left(\begin{array}{c} n\\ 2 \end{array}\right)-1\right)=\\ =\left(\begin{array}{c} n\\ 2 \end{array}\right)\left(2^{n}-2\right) \end{array}$

r:	$\begin{array}{c} \left(2^{n}-1-\left(\begin{array}{c} n\\ r \end{array}\right)\right)\left(\begin{array}{c} n\\ r \end{array}\right)+\left(\begin{array}{c} n\\ r \end{array}\right)\left(\left(\begin{array}{c} n\\ r \end{array}\right)-1\right)=\\ =\left(\begin{array}{c} n\\ r \end{array}\right)\left(2^{n}-2\right) \end{array}$

Using the equations on Table 3 we can estimate the number of all possible combinations of allocations, represented as binary strings, of hamming distance from 1 to *r*

Here *n *is the number of pathways the gene is a member of and *r *is the hamming distance. Equation (9) demonstrates that if we summate from 1 to *n*, we get the number of possible combinations corresponding to all possible hamming distances.

(9) $$\begin{array}{c} \underset{\text{Table 1, hamm}=\text{0}}{\underset{︸}{2^{n}-1}}+\underset{\text{Table 1, hamm}=\text{1,}...\text{,}n}{\underset{︸}{\overset{n}{\underset{r=1}{\sum }}\left(\begin{array}{c} n\\ r \end{array}\right)\left(2^{n}-2\right)}}\\ =2^{n}-1+\left(2^{n}-2\right)\underset{=2^{n}-1}{\underset{︸}{\overset{n}{\underset{r=1}{\sum }}\left(\begin{array}{c} n\\ r \end{array}\right)}}\\ =2^{n}-1+\left(2^{n}-2\right)\left(2^{n}-1\right)\\ =2^{n}-1+\left(2^{n}-1\right)\left(2^{n}-1\right)-2^{n}+1\\ =\left(2^{n}-1\right)\left(2^{n}-1\right) \end{array}$$

In the context of this text, we work with allocations of more than one expressed multi-membership genes to their pathways. This however, is still possible following the above rationale. Again in the simplest case of two genes, members of two pathways each, we can estimate the probability of obtaining all possible hamming distances using Table 2 and applying simple addition and multiplication of the values as shown on Table 4. Each pair of hamming distances is added to obtain the combined hamming distance, while each pairs' corresponding probability is multiplied to obtain the probability of observing the combined hamming distance in question.

**Table 4:** Combined Hamming distance and probability for a pair of genes, members of two pathways

Hamming/ Probability	0/0.333	1/0.444	2/0.222
0/0.333	0(0+0)/ 0.111(0.333 × 0.333)	1(1+0)/ 0.148(0.444 × 0.333)	2(2+0)/ 0.074(0.222 × 0.333)

1/0.444	1(0+1)/ 0.148(0.333 × 0.444)	2(1+1)/ 0.197(0.444 × 0.444)	3(2+1)/ 0.987(0.222 × 0.444)

2/0.222	2(0+2)/ 0.074(0.333 × 0.222)	3(1+2)/ 0.987(0.444 × 0.222)	4(2+2)/ 0.049(0.222 × 0.222)

Table 4 exemplifies how to estimate the combined hamming distance for two multi-membership genes, members of two distinct biochemical pathways each, along with the respective combined probability. Here again, we assume that any configuration, where each gene is allocated to at least one pathway is valid and that each one is equally likely to occur by chance.

For any number of *N *genes we can obtain the corresponding values using an *N *dimensional matrix like the above. As the number of genes grows this becomes computationally expensive, however the problem is circumvented, as each gene can be added at a sequential step, through a process of merging and expanding the matrix. For example merging the data for the two genes represented on Table 4 gives rise to the matrix on Table 5.

**Table 5:** Compact Hamming distance and probability for two genes, members of two pathways each

Hamming Distance	0	1	2	3	4
**Probability **	0.111	0.296	0.345	0.197	0.049

Table 5 is produced by merging Table 4, to only show each possible hamming distance and the corresponding probability of observing it by chance, for a set of two expressed multi-membership genes. Each gene is a member of two distinct biochemical pathways.

### Fuzzy Adjusted Rand Index

The adjusted rand index (ARI) is a common measure of crisp cluster similarity, which has been extended to fuzzy clustering giving the fuzzy adjusted rand index (FARI) [21]. For each pair of elements FARI examines if both clustering arrangements have placed the pair in the same or different clusters. We have adopted FARI to compare allocations of multi-membership genes produced by separate runs of our algorithms on the same microarray dataset, given that each arrangement may place a gene in one or more pathways. For our purposes clusters correspond to pathways, assuming equal weights for the contribution of a gene to all its member pathways.

While the hamming distance between two multi-membership gene allocations reveals biological similarity, answering the question of how similar two allocations are the fuzzy adjusted rand index examines if each pair of genes is placed together or in different pathways by subsequent runs of our algorithms.

We use both methods as two allocations may have the same fitness but different hamming distance. This can occur in cases where groups of genes are placed together but in different pathways by separate runs of our scripts. In particular for allocations of the same or very similar fitness, accompanied by significant hamming distance, high FARI value can reveal the occurrence of the above described phenomenon.

## Results and Discussion

### Hypothesis Validation

While single-membership genes through their products are solely contributing to the function of a particular pathway, multi-membership genes can participate in the functionality of any combination of the pathways they are members of, at any particular time. Thus, unlike single-membership genes, multi-membership genes' intensity values, as extracted from a microarray slide, represent a net effect. The biological system may require activation of certain pathways and regulate the production of a protein part of their network in a way that its quantity increases. At the same time it may require deactivation of other pathways in which the same protein participates. The resulting balance may affect the expression observed on the microarray leading to less consistent readings for groups of proteins part of a biochemical pathway, encoded by multi-membership genes, when each pathway is examined in isolation from the rest.

To examine this we have identified 19 experiments (GSM99081 to 83, GSM99108 to 112, and GSM99171 and GSM99172) on Escherichia coli, from microarray data available at Gene Expression omnibus (GEO) [25], platform GPL3503 [26], that contain a large number of expressed Urea Cycle genes. KEGG Urea Cycle pathway consists of 16 single-membership and 12 multi-membership genes. We divide the intensities, separately for the group of single- and the group of multi-membership genes, per experiment by their sum, to obtain a measure of the contribution of each gene to the behaviour of the pathway. We then compare the correlation between the obtained contribution values of the 12 multi-membership genes and the 16 single-membership genes, throughout the 19 experiments. For both cases we acquire a set of 171 correlation values, and perform a two sample t-test which reveals that the values are significantly different with a *p*-value of 1.3251 × 10^-12^. Furthermore, in the case of single-membership genes the correlation values are higher with 86.5% of the values being above the level of significant correlation at *p *= 1%. In contrast, for the multi-membership genes only 41.5% of the values exceed the threshold of significance at 1%. The assumption that multi-membership genes expression is the net effect of their contribution to their constituent pathways is in agreement with our findings. Single membership genes apparently show more consistent behaviour as they only contribute to the functionality of one pathway.

### Data processing with Hill climbing

In this section we perform a more detailed biological validation of some of the results produced by the hill climbing method. As the next section will show we do not to repeat this analysis using the simulated or genetic algorithm methods, as the results are virtually identical.

**Figure 2:**
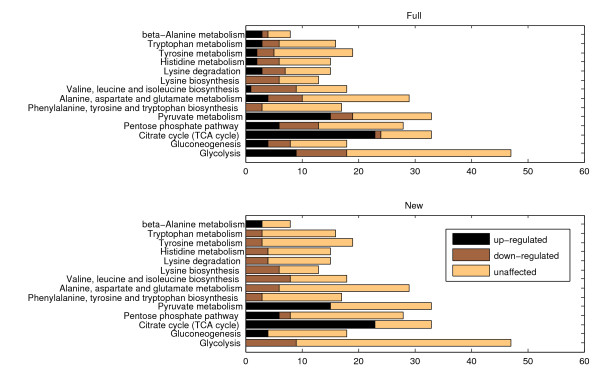
**Full membership and Hill climbing produced allocation**. The figure reveals the number of up-, down-regulated and stable genes in each of the sample pathways before and after processing. In the first case (top) entitled full, we assume that multi-membership genes are functional in all pathways of which they are members, while in the second case (bottom) the data has been subjected to processing with the hill climbing search method.

Figure 2 represents the result of applying the hill climbing search to process a popular dataset from diauxic shift experiments on *Saccharomyces cerevisiae *[27], for a set of biochemical pathways. Yeast cells inoculated in glucose rich medium turn to aerobic utilization of ethanol produced during fermentation, upon exhaustion of the available sugar. It is worth noting that KEGG includes both glycolysis and gluconeogenesis in one single pathway, as they share a number of common genes and a substantial part of each process is effectively a reversal of the other. Nevertheless, some genes are unique to glycolysis while others to gluconeogenesis and the two are never functional simultaneously, thus the two pathways have been separated to improve the efficiency of our analysis.

The first allocation on Figure 2 (top) corresponds to changes occurring in the expression of genes following the diauxic shift and represents the pathway state observed when all multi-membership genes are considered active in all pathways they participate in, according to commonly used visualisation approaches. Evidently, most pathways contain both up- and down-regulated genes. Pathways including Glycolysis, Gluconeogenesis, the Pentose phosphate pathway and Pyruvate metabolism contain similar numbers of both up- and down-regulated genes, which makes it difficult to infer their state.

As the second allocation on Figure 2 (bottom) reveals, processing of the data with our hill climbing method changes the picture substantially. As expected, upon depletion of glucose the glycolysis pathway is suppressed while expression is shifted in favour of gluconeogenesis. Rather than towards Pyruvate, reactions flow towards the biosynthetic precursor glucose-6-biphosphate which is channelled accordingly to supply the TCA cycle and gluconeogenesis. The Pyruvate metabolism pathway contains only up-regulated genes, while amino acid metabolic pathways such as the valine, leucine, isoleucine and methionine biosynthetic pathways are clearly repressed, in agreement with [28]. This is to be expected given the caloric restriction as the production of methionine is costly from a metabolic point of view, while valine, leucine and isoleucine are the most abundant amino acids in the cell.

The unique up-regulated gene in the valine, leucine and isoleucine biosynthetic pathways, LEU4, has been reallocated to the Pyruvate metabolism of which it is also a member, a pathway positively affected during the diauxic shift in agreement with [27] and [28]. All down-regulated genes in gluconeogenesis have been allocated to glycolysis where they also participate. For the unique down-regulated gene ALD6 in the beta-alanine pathway, which the authors in [28] consider one of the 15 most positively affected pathways by the diauxic shift, our method implies that the observed down-regulation may well be due to involvement of the gene in other pathways. This may be due to the involvement of ALD6 in glycolysis, phenylalanine, tyrosine, tryptophan biosynthesis and other pathways exhibiting decreased activity.

Overall, the algorithm has been able to allocate genes to pathways in a way that allows us to infer the state of individual pathways with increased certainty removing contradictions from the final results. Pathways are now mostly filled with genes of similar expression, which we consider to be the most indicative of a pathway's state.

To further investigate the results of data processing with our methodology we have applied it to *Escherichia coli *K-12 data from [29], available as experiment GSM513 at GEO. *Escherichia coli *cells were grown in tryptophan enriched medium, leading to increased activity of the tryptophan metabolism pathway. Most tryptophan metabolism genes show subtle to substantial up-regulation except from yqeF which shows significant down-regulation (Table 6). Our method has removed the down-regulated gene from the latter pathway, ascribing its behaviour to the activity of other amino acid degradation pathways, which is biologically meaningful, given that the cell is presented with excess tryptophan to partly cover its nutritional needs. In both cases discussed here, our method produces results that are consistent with the findings of the publications accompanying the data, while reducing the number of genes per pathway contradicting each other's' expression and thus allowing us to infer the state of those pathways with higher degree of confidence. The ability of this kind of approach to produce such consistent results and to substantially increase gene expression agreement per pathway seems interesting in itself. It adds some further evidence to the initial hypothesis that multi-membership gene expression represents a net effect, in the sense that the biological system regulates the expression of these genes to accommodate its need through the adequate function of the pathway they participate in.

**Table 6:** Log2 ratios of tryptophan metabolism genes, for experiment GSM513

Gene Symbol	Log2 ratio	Gene Symbol	Log2 ratio
**atoB**	1.1150	**trpS**	5.8490

**yqeF**	-1.8120	**katE**	-0.4370

**fadB**	2.6340	**katG**	1.4110

**sucA**	1.8200	**tynA**	-0.7870

**tnaA**	1.4660		

All genes showing significant differential expression are up-regulated with the exception of YqeF.

### Statistical evaluation of functional agreement

In pathway based microarray analysis, to validate data quality and identify those pathways most affected by the experimental conditions, it is common practice to estimate the probability per pathway of observing as many or more differentially expressed genes purely by chance. For example, in [28] the authors describe Pathway Processor, a tool that can be used to score biochemical pathways according to the probability that as many or more genes in a pathway would be significantly altered in a certain experiment by chance alone. Pathways accompanied by very low probability are considered more likely to be affected by the experimental conditions.

Similar approaches have been adopted to identify interesting groupings produced by cluster analysis of gene expression data. In [30] the authors use the hypergeometric distribution for the categorisation produced by clustering, to model the probability of observing at least *k *genes from a cluster of size *n *in a category of size *C *from a total genome size of *G *genes. In this way they obtain p-values (equation 10), allowing them to examine if a cluster is enriched with genes from a particular functional category to a greater extent than would be expected by chance. Clusters in which the majority of genes belong to a certain category produce low probability with *p*-values near 0.

(10) $$p=1-\overset{k-1}{\underset{i=0}{\sum }}\frac{\left(\frac{C}{i}\right)\left(\frac{G-C}{n-i}\right)}{\left(\frac{G}{n}\right)}$$

**Figure 3:**
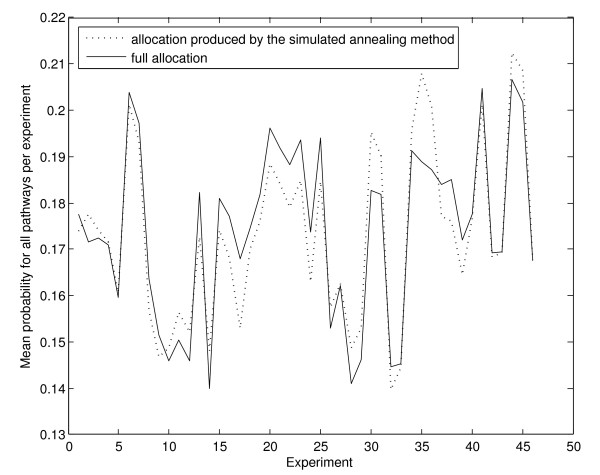
**Mean probability of observing the gene expression in hand, per pathway and experiment by chance**. The mean values per experiment are estimated upon full allocation of multi-membership genes to pathways (sold line) and processing of the dataset with our method (dotted line).

We have applied a similar approach to a microarray dataset, consisting of experiments from GEO platform 17 [31], to compare the probability of the standard full membership gene allocation to the probability of observing the allocations produced by processing of the data with the described algorithms. For our purposes, given the overall number of genes and the overall number of affected genes on the array, we obtain the probability of observing at least as many or more affected genes in a pathway of a certain size, purely by chance. Figure 3 shows the mean probability of obtaining the results in hand, per experiment. Evidently, there is no substantial change in probability, between our and the full membership allocation. However, our methodology adds an intuitional, biologically meaningful step to the analytical process.

### Methods' Performance on pathway manipulation

**Figure 4:**
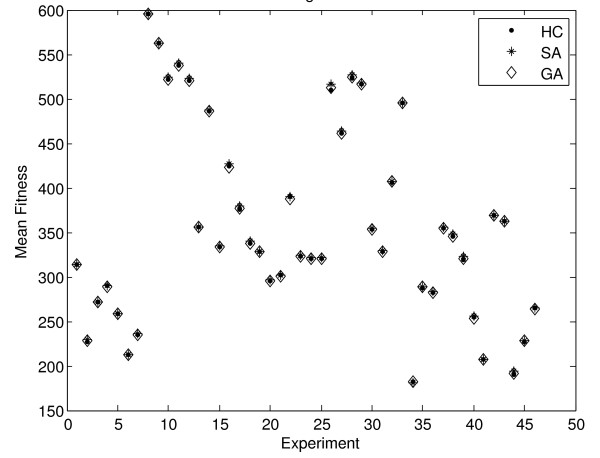
**Mean fitness reached by each method, per experiment for GPL17**. The figure reveals the mean fitness reached by the hill climbing, simulated annealing and genetic algorithm methods per experiment, for 46 experiments corresponding to platform GPL17 from GEO.

The result of implementing the hill climbing, simulated annealing and genetic algorithm to a set of 46 microarray experiments from GEO platform GPL17 is shown on Figure 4. Interestingly, all methods seem to perform quite similarly in terms of fitness. In most cases the simulated annealing approach is able to reach slightly higher fitness values. However, the difference is only subtle with a two sample t-test revealing no significant difference between the values corresponding to each search method. This is summarised on Table 7, which shows the mean of the minimum, maximum and mean fitness reached for the entire set of 46 experiments, upon ten separate runs of each method.

**Table 7:** Mean of the minimum, maximum and mean fitness reached by each method for GPL17

Hill Climbing			Simulated Annealing			Genetic Algorithm		
**Max.**	**Min.**	**Mean**	**Max.**	**Min.**	**Mean**	**Max.**	**Min.**	**Mean**

357.5	354.0	356.1	359.0	355.0	357.4	357.8	353.9	356.1

The table summarises the minimum, maximum and mean fitness reached by the hill climbing, simulated annealing and genetic algorithm method, for the entire set of 46 experiments, from GPL17.

**Figure 5:**
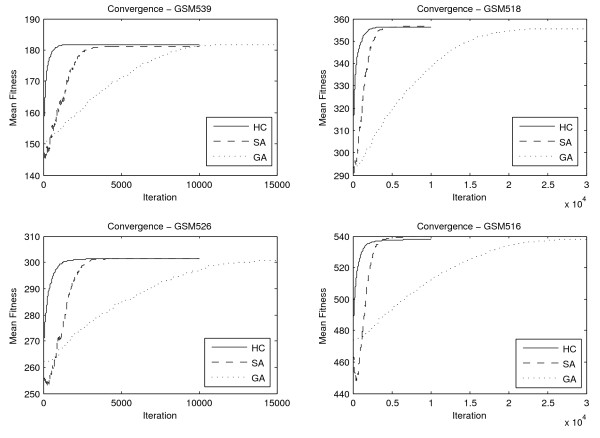
**Convergence per method for a subset of 4 microarray experiments**. The solid line corresponds to the mean hill climbing fitness reached at each iteration, the dashed line to the simulated annealing and the dotted line to the genetic algorithm. Experiments were chosen to roughly cover the range of fitness values reached in all microarray experiments. Experiments GSM539 and GSM516 where the ones with the least and most possible allocation positions and thus the ones producing the smallest and largest fitness values in the dataset respectively. Fitness values for experiments GSM518 and GSM526 are equally distanced from the two extremes.

Figure 5 represents a visualisation of the Convergence of each optimisation method for a subset of four microarray experiments. The experiments were chosen based on the mean fitness reached by 10 separate runs of each search approach, in order to exemplify the entire range of fitness values reached for GPL17. In particular the mean fitness values reached for each experiment where sorted in ascending order. GSM539 corresponds to the lower mean fitness reached for an experiment in the dataset, GSM516 to the highest mean fitness and GSM526 and GSM518 to values equally distanced from these two extremes. Evidently, the genetic algorithm approach is slower than the other methods, requiring a significantly larger number of fitness calls to converge. The hill climbing and simulated annealing methods are roughly equally efficient, with the hill climbing being slightly faster, while the simulated annealing being able to reach slightly higher fitness values, in experiments with large number of expressed multi-membership genes and thus larger search space. Naturally, as the search space grows larger, due to a larger number of expressed multi-membership genes or growing number of pathways to which such genes can be assigned, the algorithms require more iterations to converge.

**Figure 6:**
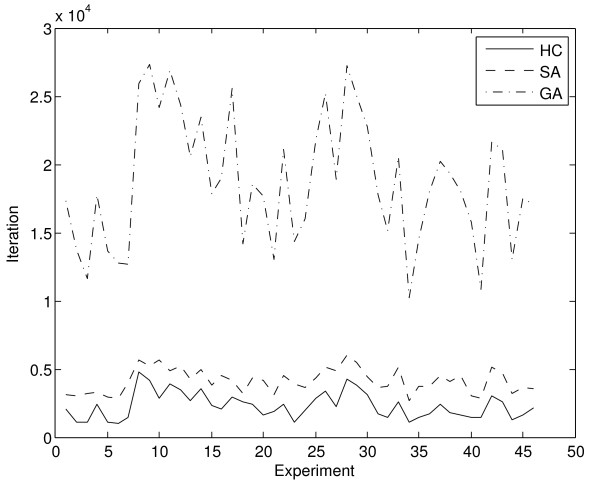
**Mean convergence per experiment and method**. The hill climbing method (solid line) is the fastest, closely followed by the simulated annealing (dashed line) approach, while the genetic algorithm (dotted line) proves significantly slower.

**Figure 7:**
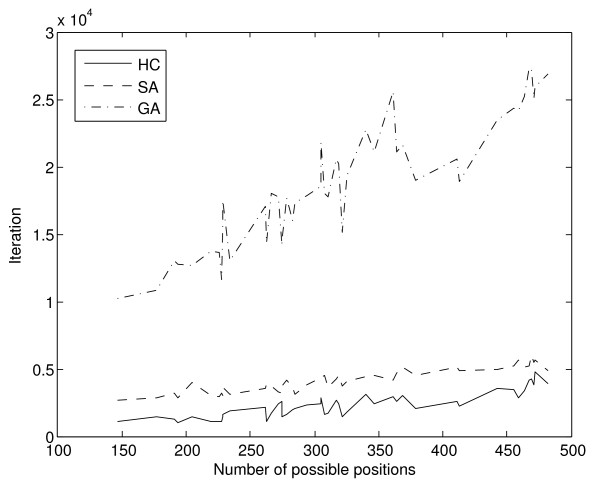
**Mean convergence per experiment, according to search space size**. As in Figure 6, Figure 7 represents the mean convergence per method and experiment. However, in this case experiments are represented in an ordered fashion, from the one with least expressed multi-membership genes, and smallest search space to the one with most expressed multi-membership genes and largest search space.

Figure 6 shows the mean number of iterations required for the algorithms to converge, while Figure 7 represents the same data in an ordered fashion from the experiment with the least number of expressed multi-membership genes and possible positions for gene allocation to the experiment with most such positions.

**Figure 8:**
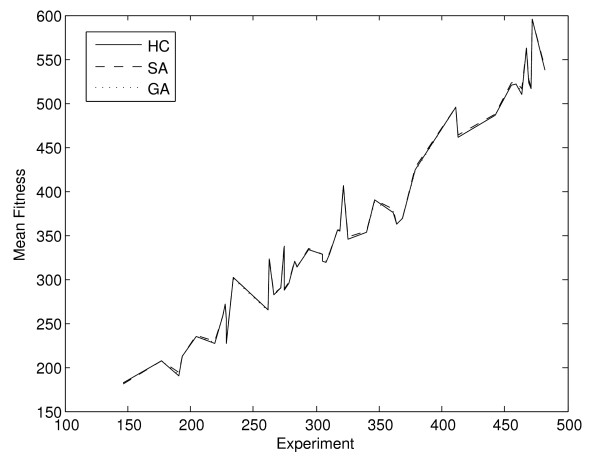
**Mean fitness per experiment and method, according to search space**. As the size of the search space grows, following the number of possible genes to pathways allocations, the methods are able to reach higher fitness values.

As expected, the mean fitness value also shows an increase as the number of allocations of genes to pathways grows (Figure 8), with highly significant correlation value of 0.9769, 0.9777 and 0.9770 for the hill climbing, simulated annealing and genetic algorithm respectively. The number of allocations of genes to pathways is determined by the number of expressed genes and the number of pathways in which the expressed multi-membership genes participate.

**Figure 9:**
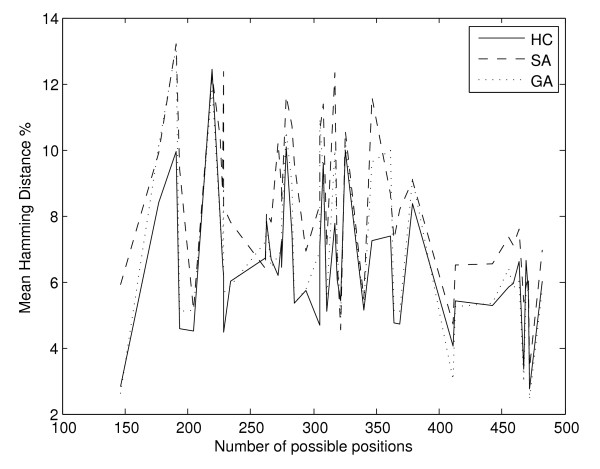
**Mean hamming distance between allocations per experiment, according to number of possible genes to pathways allocations**. Experiments are ordered according to the number of possible genes to pathways allocations. There appears to be very small correlation between the mean hamming distance in terms of percentage between separate runs of each search method and the size of the search space.

On the contrary, there is no significant correlation between the number of gene to pathways allocations and the mean hamming distance between allocations produced by subsequent runs of the three search algorithms, as shown on Figure 9. In this case we observe small correlation values of -0.2687, -0.4686 and -0.3559 for the hill climbing, the simulated annealing and genetic algorithm respectively.

**Figure 10:**
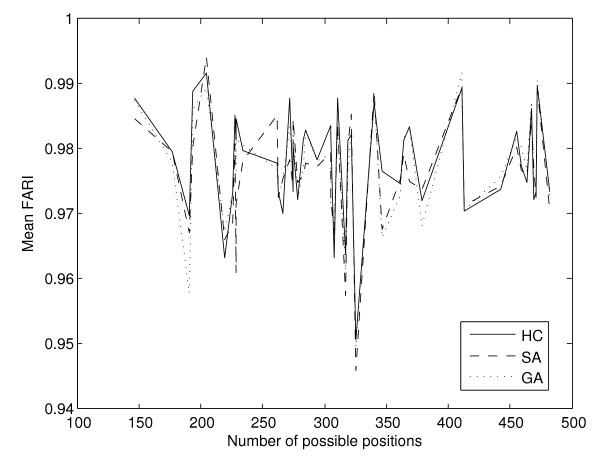
**Mean FARI between allocations per experiment, according to number of possible genes to pathways allocations**. For the FARI values between allocations produced by subsequent runs of each method there is no correlation whatsoever with the size of the search space, as defined by the number of possible multi-membership genes' allocations.

The same is true for the FARI's (Figure 10) where the correlation between the mean FARI per experiment and the number of possible multi-membership gene to pathway allocations is -0.0802, 0.0499 and 0.0308 for the hill climbing, simulated annealing and genetic algorithm respectively. Nevertheless, FARI values are extremely high for allocations produced by separate runs of each of the search techniques, as summarised on Table 8.

**Table 8:** FARI statistics between allocations produced by 10 separate runs of each search method

	Maximum	Minimum	Mean	Standard Deviation
**Hill Climbing**	1.000	0.928	0.978	0.012

**Simulated Annealing**	1.000	0.902	0.977	0.013

**Genetic Algorithm**	1.000	0.926	0.977	0.011

The table summarises the minimum, maximum and mean Fuzzy Adjusted Rand Indexes between allocations produced by ten separate runs of the hill climbing, simulated annealing and genetic algorithm search approaches.

The minimum FARI observed is 0.902, and the values remain high regardless of the observed variation in hamming distance. For example the mean FARI for pairs of allocations of hamming distance above 1 standard deviation is 0.964, 0.962 and 0.963 for the hill climbing, simulated annealing and genetic algorithm respectively. Based on this observation we can assume with sufficient degree of confidence that in cases of pairs of allocations, exhibiting substantial hamming distance, groups of genes have still been allocated together, in the same pathway, thus the FARI values are high. However, the pathways have been swapped, explaining the higher hamming distance values.

### Work on KEGG modules

While the results discussed in the data processing with hill climbing section seem biologically meaningful, a certain issue arises that is worth consideration. In particular, it can be argued that since a gene can be assigned to any of its member pathways without removal from another, the allocation in the case of each individual pathway does not affect the rest. In that sense one can examine each pathway in isolation, considering the majority of genes, in terms of up- and down-regulation. In this case the maximisation problem is reduced to a few simpler maximisation problems whose solutions can then be combined.

This approach, however, has its own drawbacks which need to be considered. Importantly, it is not possible to apply this rationale in cases where up and down-regulated genes are present in equal or even similar numbers in a pathway, such as the case of gluconeogenesis and the pentose phosphate pathway in the discussed diauxic shift experiment. In fact, examining the dataset from GPL17 discussed in the methods performance on pathways section we observed that this situation occurs in four pathways on average in each experiment. Furthermore, the condition that a gene cannot be removed from all its pathways cannot be met. By implementing the removal of the genes from each pathway that contradict the expression of the majority, we have observed that, on average, about 30 genes remain unassigned, corresponding to about 20% of all expressed multi-membership genes per experiment. Importantly, in algorithm 1, whenever this situation occurs the gene is reassigned to a pathway.

Nevertheless, the initial argument bears merit, thus we proceeded further to refine the proposed algorithmic approach. In particular, we implemented a search working with KEGG modules rather than pathways, that is, sub-networks which represent chains of events, leading to gradual alteration of a substrate into a desired product. In essence a module is still a pathway, where we zoom in to look into a particular sequence of biochemical reactions, such as the case of KEGG module M00003, representing gluconeogenesis, which forms part of the KEGG Glycolysis/gluconeogenesis pathway.

One clear advantage of working with modules is that here, in principal we expect proteins forming the module to show agreement in terms of activity, which when reflected on their respective gene expression should produce more consistent results in terms of up- or down-regulation than in the case of genes forming entire KEGG pathways.

In addition, here we can disallow allocation of expressed genes to modules of opposing nature. For example, the Glycolysis KEGG module M00001 gradually breaks down glucose to pyruvate, producing energy. In contrast, the gluconeogenesis module M00003 is responsible for the synthesis of glucose from precursors such as pyruvate. While KEGG includes glycolysis and gluconeogenesis in a single pathway due to the large number of genes shared by both, they are not simply the reverse of each other. Moreover, the two modules act in opposing directions and are not activated together as this would lead to a futile cycle [32], as previously mentioned. The same applies to amino acid and other biosynthetic and degradation modules, such as leucine, lysine and acylglycerol biosynthesis and degradation.

Hence, we have modified the algorithm to allow allocation of expressed genes shared by modules of opposing nature, which in the case of *Saccharomyces cerevisiae *applies to about 14% of multi-membership genes, to only one of these modules at any particular instance. For example whenever an up-regulated gene is assigned to glycolysis, it is removed from gluconeogenesis, as long as it is a member of both modules. We implement this at step 11) of Algorithm 1, opting for simulated annealing, previously discussed. In the following sections we present the results of implementing this approach, to which we shall refer as module algorithm, based on a number of microarray experiments. We have examined the performance of the search starting from random allocations and concentrate on results characterised by high fitness values for biological interpretation.

### KEGG modules results

The module algorithm, not allowing gene allocation to modules of opposing nature, was applied to 21 microarray experiments obtained from GEO, characterised by sufficient numbers of expressed genes and presence of genes of contradicting behaviour in the same modules. The datasets were also selected based on the experimental conditions, whose nature allows us to comment on the obtained results. Importantly, upon processing the presence of contradicting genes was reduced by 50% on average with standard deviation of about 7%. Following is a discussion of results produced by applying this approach to the aforementioned experiments, concentrating on modules where genes appear to contradict each other state of expression, especially where genes are subject to reallocation.

**Figure 11:**
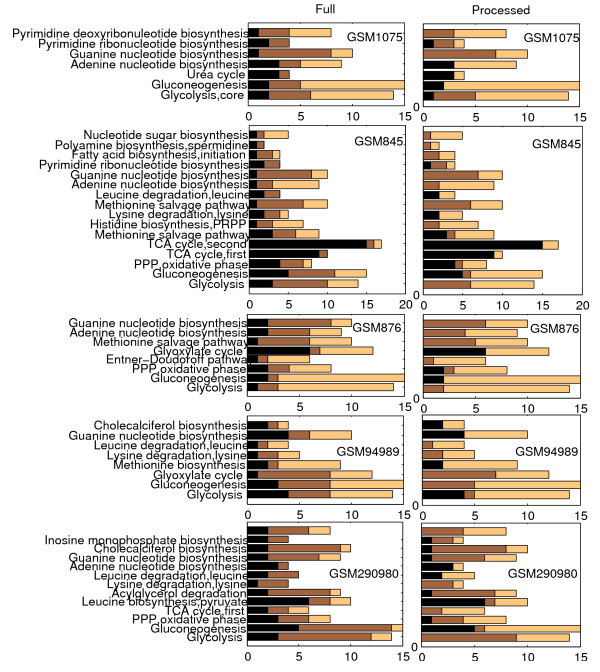
**Full membership allocation and processed allocations**. The figure reveals the number of up-, down-regulated and stable genes in each of the sample pathways, before and after processing of the data, for a number of experiments.

In GSM1075, from [33], microarray data corresponds to total RNA extracted from yeast cells subjected to adenine starvation, after 30 minutes. As observed upon processing of the data (Figure 11), the search has identified the adenine biosynthesis module as activated, while guanine biosynthesis appears supressed. We also obtain an indication that pyrimidine ribonucleotide and deoxyribonucleotide biosynthesis is supressed as the algorithm has removed up-regulated genes from the modules. At the same time glycolysis appears repressed as opposed to gluconeogenesis which has been activated.

In GSM845 yeast cells subjected to starvation, after 12 hours, exhibit activation of gluconeogenesis and suppression of glycolysis. As expected the search has removed genes from biosynthetic modules while degradation of amino acids appears activated in the case of lysine and leucine (Figure 11).

In GSM876 from the same dataset, RNA is examined 12 hours after nitrogen depletion. The researchers comment on the repressive effect of the conditions on the cluster of glycolytic genes. Indeed our algorithm has produced an allocation where Glycolysis is clearly supressed while gluconeogenesis seems in the process of activation, along with the glyoxylate cycle module (Figure 11). Nucleotide biosynthetic modules appear supressed. Results are similar one, two and three days after depletion (GSM877, 878 and 879, data not shown).

In another dataset that deals with the global response of yeast, in terms of gene expression, to glucose addition (2 g/l pulse, 15min) in the growth medium, application of the search algorithm to data corresponding to 15min following the pulse, produces allocations where up-regulated genes have been assigned to glycolysis (Figure 11). In contrast gluconeogenesis appears supressed in agreement with biological rationale. The same pattern is apparent after 20, 30, 45, 90 and 120 minutes as well as following a 0.2 g/l pulse, after 10, 15 and 20 min and in GSM 990 where glucose is once again added to the growth medium.

In contrast in GSM 290980 where RNA extracted from cells with no glucose in the medium after 2 hours are compared to cells with glucose, the search adequately assigns up-regulated genes to gluconeogenesis while down-regulated genes are allocated to glycolysis. As Figure 11 and reveals, the algorithm has reallocated a number of up-regulated genes from biosynthetic modules which is a sensible result given the condition of carbon starvation. The Entner-Doudoroff pathway which is another chain of reactions for the catabolism of glucose also appears repressed, while the leucine and lysine degradation modules are clearly activated. The picture is virtually identical after 4 hours of glucose starvation.

### Module algorithm performance

**Figure 12:**
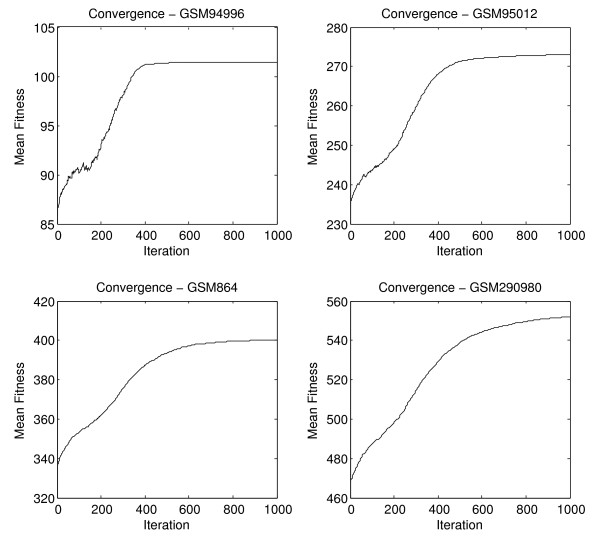
**Convergence of module algorithm**. Experiments were chosen to cover the range of fitness values reached in all microarray experiments for GPL17.

Naturally, it is worth examining the relative performance of the search based on modules, as in the case of the pathway search algorithms. Figure 12 shows the convergence of the module algorithm for four experiments based on the mean fitness reached by 10 separate runs of the search approach, in order to exemplify the entire range of fitness values.

**Figure 13:**
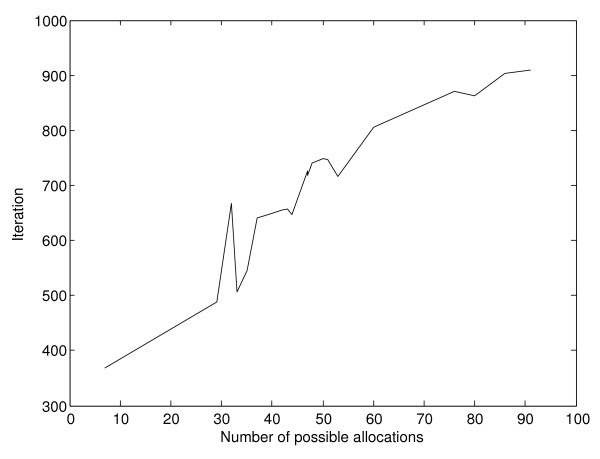
**Mean convergence of module algorithm per experiment, according to search space size**. Experiments are represented in an ordered fashion, from the one with least expressed multi-membership genes, and smallest search space to the one with most expressed multi-membership genes and largest search space.

**Figure 14:**
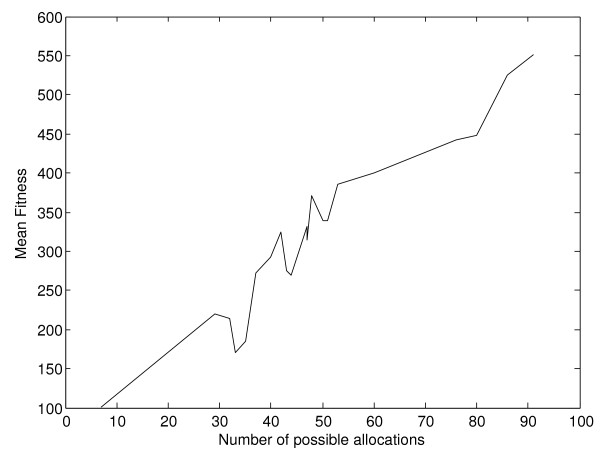
**Mean fitness per experiment for module search algorithm, according to search space**. As the size of the search space grows, following the number of possible genes to pathways allocations, the mean fitness reached by the algorithm follows suit.

**Figure 15:**
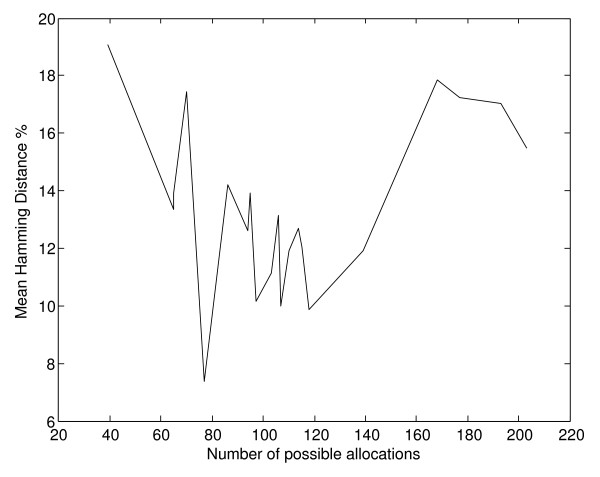
**Mean hamming distance, according to number of possible genes to pathways allocations, for module algorithm**. There appears to be very small correlation between the mean hamming distance of allocations produced by separate runs of the module search algorithm in terms of percentage and the size of the search space.

As previously observed the, the mean number of iterations required for the algorithm to converge exhibits significant correlation of 0.9419 to the number of possible gene allocations (Figure 13), as does the mean fitness with correlation of 0.9616 (Figure 14). At the same time, there is no significant correlation (0.2265) between the number of genes to modules allocations and the mean hamming distance between allocations produced by subsequent runs of the search, as exhibited on Figure 15. This situation is similar to what we observed working with KEGG pathways in the preceding sections.

**Figure 16:**
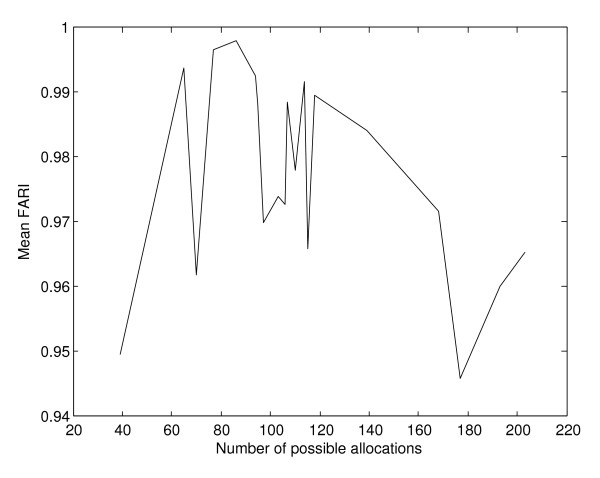
**Mean FARI between allocations, according to number of possible genes to pathways allocations, for module algorithm**. FARI values between allocations produced by subsequent runs of the method exhibit no correlation whatsoever to the size of the search space, as defined by the number of possible multi-membership genes' allocations.

The same applies to the mean FARI's, where the correlation between the mean FARI per experiment and the number of possible multi-membership gene to module allocations is -0.3700 (Figure 16). Once again we observe extremely high FARI values for allocations produced by separate runs of the search, with mean FARI of 0.9777 and standard deviation of only 0.0157. Importantly, FARI remain high even for variable hamming distances, which as in the case of gene to pathways allocations suggests that in certain pairs of allocations, exhibiting substantial hamming distance, groups of genes have still been allocated together, in the same module, leading to high FARI values.

## Conclusions

We have shown that our algorithms can effectively assign multi-membership genes to their constituent pathways and modules, increasing the level of agreement, in terms of the direction of expression in either case. Nevertheless, working with modules seems advantageous from both biological and analytical point of view. That is, genes in a module are expected to show more consistent behaviour, while the more detailed definition of biochemical processes forming modules allows us to identify modules of opposing nature. In such cases we restrict allocation of the same expressed genes to both processes.

The methodology is of potential interest, in the effort to infer the state of individual pathways and modules  based on microarray data analysis. It suggests an interesting direction for future work, as the multi-membership nature of genes has not been extensively considered as such in relevant research.

Interestingly, we have observed minimal variation in the performance of the three search approaches, namely the hill climbing, simulated annealing and genetic algorithm. All methods produce highly consistent results and reach roughly equal fitness values, although the simulated annealing approach does seem slightly superior. Furthermore, the consistency of the produced allocations in terms of Hamming distance and FARI values does not show any correlation to the size of the search space, as defined by the number of possible genes to pathways or modules allocations in each experiment.

A related issue that may be resolved following this approach is the observed swapping of piles of genes between pathways, by subsequent runs of the search algorithms. As discussed in the preceding sections, certain groups of genes seem to be allocated to different biochemical processes, but still placed together by separate applications of the methods described here. There is room for further investigation in that respect.

An issue that requires more thorough investigation is the presence of genes, e.g. repressors, for which it is expected to observe change in expression that contradicts the up- or down-regulated state of the pathway they are members of. While our methods can still produce meaningful results, since this is confined to individual cases, especially when modules are used for the analysis, we plan to tackle this issue by improving our fitness function, taking into account the behaviour of suppressors and facilitating a more detailed pathway categorisation, for example using the Reactome database.

It is worth noting that the methodology has been applied to *Escherichia coli *and *Saccharomyces cerevisiae *which are relatively simple living forms. According to KEGG statistics, the number of protein genes found in *Escherichia coli *K-12 is 4149 with 1397 of them allocated to biochemical pathways. In contrast, a human cell encloses 25724 protein genes, 5283 of which have currently been allocated to 198 KEGG pathways. Due to the resulting larger search space the methodology discussed here is likely to generate greater reshuffling in the results produced upon processing of microarray data from such more sophisticated organisms.

Finally, an appealing direction for future work would be the use of the proposed approach on a large dataset, consisting of thousands of microarray experiments to infer the state of individual pathways in terms of activation and subsequently apply association rules mining between biochemical processes in an effort to elucidate pathway regulation and interaction.

## Competing interests

The authors declare that they have no competing interests.

## Authors' contributions

All authors contributed to the development of the underlying concept; SP wrote the scripts and was the principal researcher; AP aided in interpretation; SS aided software development. All authors read and approved the final manuscript.

## References

[B1] Schena M Shalon D Davis R Brown PO Quantitative monitoring of gene expression patterns with a complementary DNA microarray Science 1995 270 467 470 10.1126/science.270.5235.467 7569999

[B2] Cavalieri D Castagnini C Toti S Maciag K Kelder T Gambineri L Angioli S Dolara P Eu.Gene Analyzer a tool for integrating gene expression data with pathway databases Bioinformatics 2007 23 19 2631 2632 10.1093/bioinformatics/btm333 17599938

[B3] Kanehisa M Goto S Kyoto encyclopaedia of genes and genomes Nucl Acid Res 2000 28 27 30 10.1093/nar/28.1.27 PMC102409 10592173

[B4] Joshi-Tope G Gillespie M Vastrik I D'Eustachio P Schmidt E de Bono D Jassal B Gopinath GR Wu GR Matthews L Lewis S Birney E Stein L Reactome: a knowledgebase of biological pathways Nucl Acid Res 2005 33 Database D428 D432 10.1093/nar/gki072 PMC540026 15608231

[B5] Rojas I Golebiewski M Kania R Krebs O Mir S Weidemann A Wittig U SABIO-RK: a database for biochemical reactions and their kinetics BMC Systems Biology 2007 1 Suppl 1 S6 10.1186/1752-0509-1-S1-S6

[B6] Karp PD Riley M Paulsen IT Collado-Vides J Paley SM Pellegrini-Toole A Bonavides C Gama-Castro S The EcoCyc Database Nucl Acid Res 2002 30 1 56 58 10.1093/nar/30.1.56 PMC99147 11752253

[B7] Dahlquist KD Salomonis N Vranizan K Lawlor SC Conklin BR GenMAPP, a new tool for viewing and analyzing microarray data on biological pathways Nat Genet 2002 31 1 19 20 10.1038/ng0502-19 11984561

[B8] Shannon P Markiel A Ozier O Baliga NS Wang JT Ramage D Amin N Schwikowski B Ideker T Cytoscape: a software environment for integrated models of biomolecular interaction networks Genome Research 2003 13 11 2498 504 10.1101/gr.1239303 14597658 PMC403769

[B9] Goesmann A Haubrock M Meyer F Kalinowski J Giegerich R PathFinder: reconstruction and dynamic visualization of metabolic pathways Bioinformatics 2002 18 124 9 10.1093/bioinformatics/18.1.124 11836220

[B10] Kolpakov FA Ananko EA Kolesov GB Kolchanov NA GeneNet: a gene network database and its automated visualization Bioinformatics 1998 14 529 537 10.1093/bioinformatics/14.6.529 9694992

[B11] Toyoda T Mochizuki Y Konagaya A GSCope: a clipped fisheye viewer effective for highly complicated biomolecular network graphs Bioinformatics 2003 19 437 8 10.1093/bioinformatics/btg001 12584139

[B12] Panteris E Swift S Payne A Liu X Mining pathway signatures from microarray data and relevant biological knowledge Journal of Biomedical Informatics 2007 40 6 698 706 10.1016/j.jbi.2007.01.004 17395545

[B13] Stryer L Berg MJ Tymoczko LJ Biochemistry 2002 5 W.H 612 Freeman

[B14] Quadroni M James P Proteomics and automation Electrophoresis 1999 20 664 677 10.1002/(SICI)1522-2683(19990101)20:4/5<664::AID-ELPS664>3.0.CO;2-A 10344232

[B15] Greenbaum D Colangelo C Williams K Gerstein M Comparing protein abundance and mRNA expression levels on a genomic scale Genome Biology 2003 4 9 117 10.1186/gb-2003-4-9-117 12952525 PMC193646

[B16] Russo G Zegar C Giordano A Advantages and limitations of microarray technology in human cancer Oncogene 2003 22 6497 6507 10.1038/sj.onc.1206865 14528274

[B17] Pavlidis S Payne A Swift S An Improved Methodology for Pathway Based Microarray Analysis Based on Identification of Individual Pathways Responsible for Gene Regulation IDAMAP 2008 69 74

[B18] Russell SJ Norvig P Artificial Intelligence: A Modern Approach 2003 2 Upper Saddle River, NJ: Prentice Hall

[B19] Kirkpatrick S Gelatt CD Jr Vecchi MP Optimization by simulated annealing Science 1983 220 671 680 10.1126/science.220.4598.671 17813860

[B20] Holland JH Adaptation in Natural and Artificial Systems: An Introductory Analysis with Applications to Biology, Control, and Artificial Intelligence 1975 Ann Arbor, MI: University of Michigan Press

[B21] Brouwer RK Extending the rand, adjusted rand and jaccard indices to fuzzy partitions Journal of Intelligent Information Systems 2009 32 213 235 10.1007/s10844-008-0054-7

[B22] Blattner FR Plunkett G Bloch CA Perna NT Burland V Riley M Collado-Vides J Glasner JD Rode CK Mayhew GF Gregor J Davis NW Kirkpatrick HA Goeden MA Rose DJ Mau B Shao Y The Complete Genome Sequence of *Escherichia coli *K-12 Science 1997 277 5331 1453 1462 10.1126/science.277.5331.1453 9278503

[B23] Michalewicz Z Fogel DB How to solve it: Modern heuristics 1998 Berlin: Springer

[B24] Hamming R Error Detecting and Error Correcting Codes Bell System Technical Journal 1950 26 2 147 160

[B25] Barrett T Troup DB Wilhite SE Ledoux P Rudnev D Evangelista C Kim IF Soboleva A Tomashevsky M Edgar R NCBI GEO: mining tens of millions of expression profiles--database and tools update Nucleic Acids Res 2006 35 Database D760 D765 10.1093/nar/gkl887 PMC1669752 17099226

[B26] Gene Expression Omnibus Platform GPL3503 http://www.ncbi.nlm.nih.gov/geo/query/acc.cgi?acc=GPL3503

[B27] DeRisi JL Iyer VR Brown PO Exploring the metabolic and genetic control of gene expression on a genomic scale Science 1997 278 680 686 10.1126/science.278.5338.680 9381177

[B28] Grosu P Townsend JP Hartl DL Cavalieri D Pathway processor: a tool for integrating whole-genome expression results into metabolic networks Genome Res 2002 12 1121 1126 10.1101/gr.226602 12097350 PMC186628

[B29] Khodursky AB Peter BJ Cozzarelli NR Botstein D Brown PO Yanofsky C DNA microarray analysis of gene expression in response to physiological and genetic changes that affect tryptophan metabolism in *Escherichia coli* PNAS USA 2000 97 12170 12175 10.1073/pnas.220414297 11027315 PMC17313

[B30] Swift S Tucker A Vinciotti V Martin N Orengo C Liu X Kellam P Consensus clustering and functional interpretation of gene-expression data Genome Biology 2004 5 R49 10.1186/gb-2004-5-7-r49 15535870 PMC545785

[B31] Gene Expression Omnibus Platform GPL17 http://www.ncbi.nlm.nih.gov/geo/query/acc.cgi?acc=GPL17

[B32] Champe PC Harvey RA Ferrier DR Lippincotts Illustrated Reviews Biochemistry 2004 3 Lippincott reverend & adventurer

[B33] Gasch AP Spellman PT Kao CM Carmel-Harel O Genomic expression programs in the response of yeast cells to environmental changes Mol Biol Cell 2000 11 12 4241 4257 11102521 10.1091/mbc.11.12.4241 PMC15070

